# Robotising vitreoretinal surgeries

**DOI:** 10.1038/s41433-024-03149-3

**Published:** 2024-07-04

**Authors:** Helen Mi, Robert E. MacLaren, Jasmina Cehajic-Kapetanovic

**Affiliations:** 1 Oxford Eye Hospital, Oxford University Hospitals NHS Foundation Trust, Oxford, UK; 2 Nuffield Laboratory of Ophthalmology, Nuffield Department of Clinical Neurosciences, University of Oxford, Oxford, UK; 3 NIHR Oxford Biomedical Research Centre, Oxford, UK

**Keywords:** Surgery, Retinal diseases, Disease genetics

## Abstract

The use of robotic surgery in ophthalmology has been shown to offer many potential advantages to current surgical techniques. Vitreoretinal surgery requires complex manoeuvres and high precision, and this is an area that exceeds manual human dexterity in certain surgical situations. With the advent of advanced therapeutics such as subretinal gene therapy, precise delivery and minimising trauma is imperative to optimize outcomes. There are multiple robotic systems in place for ophthalmology in pre-clinical and clinical use, and the Preceyes Robotic Surgical System (Preceyes BV) has also gained the CE mark and is commercially available for use. Recent in-vivo and in-human surgeries have been performed successfully with robotics systems. This includes membrane peeling, subretinal injections of therapeutics, and retinal vein cannulation. There is huge potential to integrate robotic surgery into mainstream clinical practice. In this review, we summarize the existing systems, and clinical implementation so far, and highlight the future clinical applications for robotic surgery in vitreo-retina.

## Abstract (zh)

在眼科应用机器人进行手术已证实比人工手术技术具有许多潜在优势。玻璃体视网膜手术是复杂和高精度的操作, 在某些手术情况下, 其操作的灵活性超过人类手工的操作能力。随着视网膜下治疗等先进疗法的出现, 需要精准施术并尽量减少损伤。目前有多种眼科机器人系统用于临床前期和临床应用中, Preceyes机器人手术系统 (Preceyes BV) 也获得了CE认证, 并已投入商业使用。当前手术机器人成功地完成了某些体内和人体手术, 包括膜剥离, 视网膜下药物注射, 和视网膜静脉插管。将机器人手术整合到主流临床实践中具有巨大潜力。在这篇综述中, 我们总结了现有的系统、迄今为止的临床实施, 并强调了机器人手术在玻璃体视网膜领域在临床应用的前景。

## Introduction

Microsurgery in ophthalmology demands high precision and accuracy, with a steady and dexterous surgical approach to allow for minimal tissue damage within the small confines of the eye [1]. This is especially important in certain structures such as the retina where margins of errors are low as the tissues do not regenerate and any surgical trauma may result in potentially devastating consequences [2]. The clinical integration of robotics has become common in various other surgical fields for better manoeuvrability and increased precision, and the da Vinci Surgical System (Intuitive Surgical, Inc.) has become the most prevalent surgical system in the fields of general surgery, urology and gynaecology [3]. Similarly, robotics in ophthalmic retinal microsurgery offers considerable advantages to overcome existing limitations with manually controlled instruments [4]. Robotic eye surgery was first described in 1989 [5], but since then there has been a slightly delayed transition of intraocular surgical robotic systems to routine clinical practice. This is likely attributed to the unique processes of intraocular surgery, but significant advancements and developments have been made in the past decade. The goals of this review paper are to highlight the robotic systems in development, in both pre-clinical and clinical use, recent breakthroughs with in-human studies, clinical approaches with robotic systems, challenges in integrating robotics system with mainstream clinical use, and potential exciting future applications.

## Challenges of intraocular microsurgery

Intraocular microsurgery remains challenging to perform, with concerns involving accuracy, tremors, precision, depth perception, and dexterity. In vitreoretinal surgeries, high accuracy is required in a small workspace during surgical manipulation [6]. As such, physiological hand tremors becomes a substantial concern. Previous experimental characterizations of physiologic tremors showed that the average root mean square (rms) amplitude of tremor ranged between 14 and 142 µm when holding a tool still, and between 59 and 341 µm when actuating a microsurgical grasper. Tremor data recorded during eye surgery have shown that it is present in the order of 100 µm in all directions of hand movement in its peak-to-peak excursion when transmitted to the tip of the instrument. Previous study also reported a rms amplitude of tremor at 38 µm when tracking the tool motion during epiretinal membrane peeling [7]. This results in difficulty in being absolutely precise in targeting a specific area of concern, or holding the surgical instrument stationary for prolonged durations.

This is particularly of concern in vitreoretinal surgeries, where delicate structures need to be manipulated surgically with surgical tools. Robotic technology has been demonstrated to assist in overcoming physiological tremors for more desired surgical outcomes [8]. With the advent of subretinal gene therapy, such as the recently FDA-approved gene therapy for Leber congenital amaurosis, voretigene neparvovec-rzyl (Luxturna) [9], elimination of hand tremors to allow for precise delivery with minimal surgical trauma is imperative to improve surgical safety compared to the current manual subretinal injection protocol [9]. Many ongoing clinical trials for gene therapy or stem cell therapy for inherited retinal diseases are currently going on, but precise delivery of such treatment methods is necessary for desired surgical outcomes with reduced reflux and retinotomy sizes. Certain conditions such as retinal vein occlusion which is a highly prevalent disease affecting 16.4 million people worldwide [10], still have inadequate standard of care. Currently, potentially curative treatment methods are retinal endovascular surgery [11], but this requires complex surgical manoeuvres exceeding the human skill and human hand’s stability.

Depth perception in ophthalmic microsurgery also poses another significant constraint [12]. In vitreoretinal surgeries, visualization is often challenging in view of the semitransparent features of retinal anatomy and difficulty with the perception of tool shadows due to complex lighting conditions with a chandelier illumination system or handheld endoilluminator light [13]. This can also be a challenge in anterior segment surgeries, such as limited resolution to sense the exact depth of the posterior capsule resulting in higher than necessary rates of posterior capsular rupture. For these reasons, robotic systems together with integrated imaging modalities such as intraoperative optical coherence tomography (OCT) will offer a solution to these problems.

Another challenge in intraocular microsurgery is surgical tool dexterity. Surgical tools are generally slender instruments with rigid shafts, and they are constrained to the traditional four-DOF (degree of freedom) motions [13]. This includes tilt in two directions and rotation about and translation along the longitudinal axis of the tool. This results in limited tool-tip dexterity, which may impede complex vitreoretinal surgeries especially precise work such as potential endovascular surgeries. With the development of clinical tools especially instruments of extreme small gauge calibre [14], improved instrumentation and dexterity will be necessary to fully utilize the instruments and produce more consistent outcomes with device manipulation.

## Robotics system in development

The first robotic systems in ophthalmology were developed for the anterior segment, and at the moment, they are still currently in development. However, vitreoretinal robotic systems are in more advanced stages of development with over 12 systems being developed. Most of these systems are in pre-clinical stages, but two systems are in actual clinical use. Out of the two robotic systems in the clinical stage, the Preceyes Robotic Surgical System has gained the CE mark in 2019.

The first instance of robotic ocular microsurgery was described in 2007, using the da Vinci Surgical System for corneal laceration repair with 10-0 nylon sutures in porcine models [15]. This is a console system where the surgeon is seated at the console controlling the slave arms and camera. However, although this system provided a certain level of precision without mechanical tremor, the instrumentation is precise to within 1 mm. For successful implantation in ophthalmic surgery, further precision is required to be within microns [16].

Since then, multiple robotic systems for vitreoretinal microsurgery have been developed with various principles in pre-clinical and clinical use. Amongst the systems in pre-clinical use, they include handheld devices such as the Micron system developed at Carnegie Mellon University [17], co-manipulator systems such as the Steady-Hand Eye robotic system developed at Johns Hopkins University [18], telemanipulator systems such as the intraocular robotic interventional surgical system (IRISS) developed at the University of California, Los Angeles [19], RAM!S system from the Technical University of Munich, as well as the developing telemanipulator Acusurgical system from France (based at the Montpellier Laboratory of Computer Science, Robotics and Microelectronics) [20, 21]. Intraocular magnetic robotic systems have also been described in pre-clinical use, such as microcapsular robots guided by a magnetic field system called the OctoMag [22]. There are currently two systems in clinical use, with the Preceyes Surgical System and the KU Leuven co-manipulator robotic system.

The Micron is a handheld micromanipulator device developed through a collaboration between the Robotics Institute at Carnegie Mellon University and Johns Hopkins University, which aims to reduce hand tremors, with a 90% reduction in tremors reported. The tool was evaluated by performing retinal vein cannulation on ex vivo porcine eyes and demonstrated an increased success rate from 29% to 63% compared to not using the device [17]. It was also demonstrated that an automated position-holding feature can allow for the tool tip to be maintained steady in an artificial vein for a significantly longer period of time [23]. However, all studies to date has been performed in animal models or artificial eyes.

The Steady-Hand Eye Robot is a cooperative system developed at Johns Hopkins University, where the surgeon and the robotic actuator control a surgical tool simultaneously [24, 25]. The robot controller reads force signals from the surgeon’s hands which are moving the instruments in a normal manner. This then produces a smooth motion profile while eliminating tool movements that result from hand tremors. Preliminary experiments have been carried out using vein models and artificial material [25], but the model is still in the pre-clinical, with continued optimization of the system.

The IRISS was developed with the overall goal of a robotic surgical system capable of performing anterior and posterior segment ocular surgeries [26], through augmented reality teleoperation and automation. It consists of two manipulators that mount and travel on semicircular tracks, capable of mounting any commercially available surgical instrument and switching them outside the eye in milliseconds [13]. The surgeon teleoperates the robotic system from a distance using a pair of custom joysticks, with the robot motion having motion-scaling and tremor-reduction techniques. Visual feedback is obtained three-dimensionally through a stereo camera, and displayed to the surgeon via a heads-up monitor [27]. More recently, the IRISS was evaluated by surgeons using post-mortem porcine eyes and shown to be effective in performing many key steps including performing an entire cataract extraction from start to finish [19].

RAM!S is a telemanipulator system developed by the Technical University of Munich, and works with a hybrid parallel-serial mechanism that includes piezoelectric motors for actuation [20]. This system was evaluated on ex vivo porcine eyes [28], and further work on the robotic system allowed the surgeon to perform precise and comfortable micromanipulation. More recently, subretinal depth tracking of a needle with the guidance of OCT has been performed in ex vivo pig eyes [29], providing a reference for future work.

Intraocular robotic magnets have also been reported, and these systems utilize an extraocular magnetic field to control these magnets. Robotic microcapsules within the eye have been described for procedures like retinal vein cannulation and localized drug delivery [22]. The potential advantage is achieving intraocular dexterity and manoeuvres without physical attachment to external space. The OctoMag magnetic field system [30] has been used to drive a magnetic tip microcannula for subretinal gene therapy delivery [31] and offers a potential safety advantage over traditional surgical tools due to their limited rigidity and deliverable forces. However, this system exhibits 11 degrees of angular error of the magnetic field, and the magnetic field alignment precision needs to be optimized for clinical use.

Moving onto the systems in clinical use, firstly, one system in clinical use is the KU Leuven co-manipulator robotic system, which was developed to increase the surgeon’s precision and stability by stabilizing the eye and improving precision. The eye is stabilized using a pre-operative alignment system, and surgical precision is enhanced by rendering motion-opposing forces which increase in magnitude with the speed of motion [32]. This was tested in a porcine retinal vein model, that showed that prolonged retinal vein cannulation [11] with local intravenous medication infusion of up to 10 min is possible. The first-in-human robot-assisted retinal vein cannulation was then performed in 4 patients, with injection of ocriplasmin into the targeted retinal veins of periods up to 10 min, and we await further detailed reports on the clinical outcomes of the study.

**Fig. 1:**
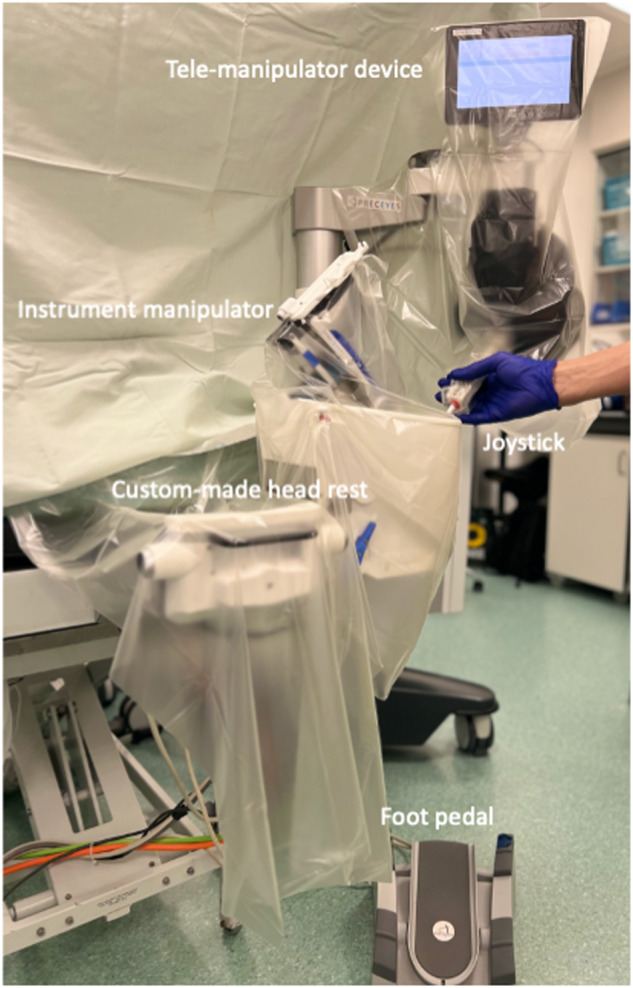
The Robotic System in clinical use: The Preceyes Robotic Surgical System (Preceyes BV). The system work as a telemanipulator device with separate joystick and manipulator.

**Fig. 2:**
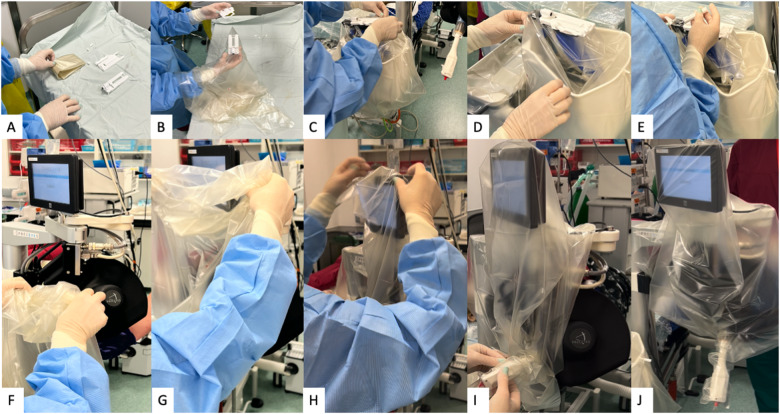
Setting up and sterile draping with the robotic system. **A**–**J** The set-up of the robotic system is performed in the operating theatre, with the surgeon initially setting up the system of the instrument manipulator and meticulous placing of the sterile draping over the robotic parts.

**Fig. 3:**
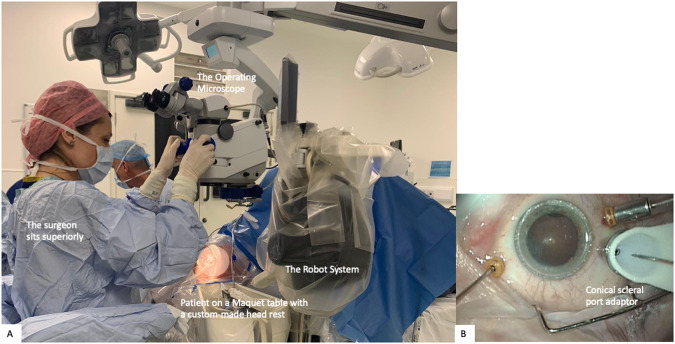
The Preceyes system set-up in the operating theatre. **A** The Preceyes system in action during an operative procedure. **B** Surgical view under the microscope. The white conical tip of the instrument manipulator is docked securely with the conical port. Advancement of the instruments necessary can be performed through the docked tip, while the surgeon holds the light pipe in the other hand.

**Fig. 4:**
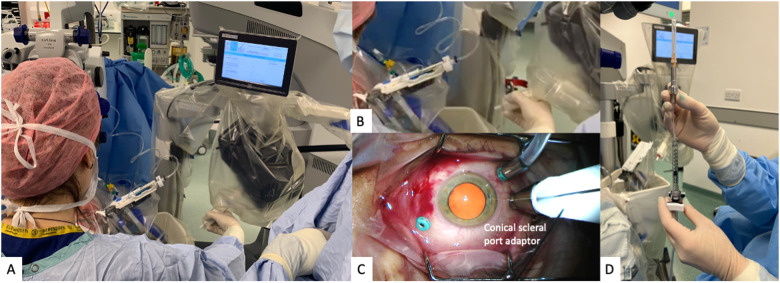
The robot system in clinical practice: Intraocular drug delivery. **A** The surgeon is holding and manipulating the motion controller mounted to the headrest, which translates into controlled movement with the instrument micromanipulator. This actual surgical positioning shows the hybrid nature of the surgery with the surgeon holding the endo-illuminator in one hand, and the other hand to control the instrument manipulator. **B** Magnified view of the motion controller held by the surgeon, and the instrument manipulator which is docked into the scleral adaptor. **C** The conical scleral adaptor is fitted over the superotemporal transscleral port for docking of the tip of the instrument manipulator. **D** The r-TPA injection delivery system which was connected via the automated viscous fluid control port of the vitrectomy machine, allowing foot-controlled steady injection of the solution under direct intraoperative OCT visualization.

The Preceyes Robotic Surgical System (Fig. 1) was developed by the Preceyes BV, a spin-off company of the Eindhoven University of Technology in the Netherlands. The device was built for vitreoretinal procedures in compliance with 93/42/EEG and has gained the CE Mark in 2019. The system has a motion controller that the surgeon uses to command surgical tool-tip position, and this can be fitted with an array of standard microsurgical instruments such as forceps and injectors [33]. The system has also been integrated with external OCT imaging intraoperatively to establish tool-tip boundaries and positioning [34]. The precision of the system has a tool-tip positional resolution of 10 µm [35]. The robotic system installation for the Preceyes system has been shown to not disrupt normal surgical workflow. The system will be set up prior to each case entering the theatre, with the telemanipulator positioned at the top of the operating table, and thereafter meticulous draping over the robotic parts was performed prior to commencement of surgery (Fig. 2). The surgeon sits superiorly and manipulates the handheld motion controller in one hand, and the endoilluminator in the other hand (Fig. 3A). The operating surgeon manipulates the handheld motion controller with direct visualization of the system under the microscope (Fig. 3B), generating large-scale movements that are translated into precise micromovements at the tip of the instrument. For most cases, after standard vitrectomy, the conical tip of the instrument manipulator was docked to a customized conical-shaped scleral port adaptor (Fig. 4C) allowing the eye to be secured firmly when instruments are in the vitreous cavity.

The first-in-human randomized controlled study using this device was conducted in patients for epiretinal or inner limiting membrane peeling. Surgical outcomes were shown to be equally successful, safe and viable in this system [36]. Subsequently, a first-in-human randomized controlled trial was also performed successfully with the Preceyes surgical system for subretinal drug delivery of tissue plasminogen activator (TPA) with intraoperative OCT [37]. Injection of the TPA was performed using the robot’s control, direct visualization and intraoperative OCT (Fig. 4A–B). The study showed that the system is safe and well tolerated, with similar surgical time and retinal microtrauma when compared to conventional manual technique. This demonstrates its potential future application in subretinal gene or cell therapy.

## Clinical applications with robotic systems

Given the mentioned advantages of robotic systems in vitreoretinal surgeries, this has been applied to multiple clinical applications in human trials. These are mainly in cases of epiretinal membrane or inner limiting membrane peeling [36], subretinal injections [37], and retinal vein cannulation [32, 33, 35].

### Membrane peeling

**Fig. 5:**
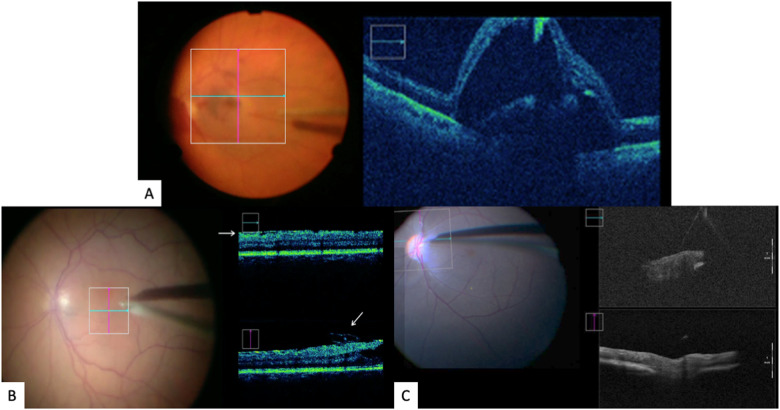
The robotic system applications: Intraoperative view. **A** Intraoperative surgeon’s view of sub-retinal drug delivery with the robot-assisted system using a 41G cannula creating a retinotomy at the macula, coupled with intraoperative OCT assistance. Slow delivery of drug in the sub-retinal space with gradual bleb formation under direct OCT visualization. **B** Robotic-assisted system for flap initiation for ERM or ILM flap lifting. Advancement of the pick and dynamic intraoperative OCT shows a flap of ERM being lifted by the pick. **C** Intraoperative view of endovascular vein cannulation using robot-assisted system with intraoperative OCT guidance.

The first-in-human study using an electronic robotic device to perform high-precision surgery was done using the Preceyes robotic system and was performed in 2018 for patients requiring epiretinal or inner limiting membrane dissection over the macula [36]. This was a double-armed randomized clinical investigation comparing robot-assisted versus traditional manual surgery in patients undergoing removal of membranes, under general anaesthesia. Visualization of the retina and intraoperative optical coherence tomography were obtained by Zeiss Rescan 700 operating microscope (Carl Zeiss Meditec AG, Jena, Germany). The robotic-assisted surgeon was compared to manual surgery alone for the step requiring maximal precision, the initiation of the flap away from the macula surface using a bevelled needle or “pick”. After standard pars plana vitrectomy, the conical tip of the instrument manipulator was docked to a customized conical-shaped scleral port adaptor (Fig. 3B) allowing the eye to be secured firmly. The pick was then advanced into the eye via the conical elements, valved port, and into the vitreous cavity. With intraoperative OCT guidance, the flap was lifted and initiated with the pick (Fig. 5B).

The results of this trial showed that although the surgical time and flap initiation time were longer in the robot-assisted group, the final anatomical outcomes were equally successful in the robot and manual control eyes with the closure of macular holes and removal of epiretinal membranes in all patients, as confirmed by spectral-domain optical coherence tomography (Spectralis, Heidelberg Engineering, Heidelberg, Germany). There were fewer iatrogenic retinal microtrauma or micro-haemorrhages in the robot cases compared to controls, and whilst this was not statistically different, this result was supportive of the robotic system’s safety profile. The results from this trial serve to demonstrate the safety and success of the Preceyes system in vitreoretinal surgery, which can then be applied to other even more delicate vitreoretinal procedures.

### Subretinal injections

In the next phase of the same trial performed in 2018 for membrane peeling, the Preceyes robotic system was subsequently used to perform subretinal injection of recombinant tissue plasminogen activator (r-TPA, Alteplase, Boehringer-Ingelheim, Germany) under local anaesthesia [36] in patients with subretinal haemorrhage. Subretinal injections were completed successfully in 2 out of 3 patients, with one of the patients developing transient intraoperative exacerbation of cataract which precluded a clear view of the cannula tip. Subsequently, another double-armed, randomized controlled trial was performed on 12 participants with acute sub-foveal haemorrhage secondary to neovascular age-related macular degeneration under local anaesthesia [37]. The setup was once again unobtrusive and did not disrupt the surgical workflow. The surgeon manipulated the handheld motion controller in 4 possible axes  (Fig. 4A–B) and thereafter guided the tip of the cannula through the scleral port fitted with a custom-made adaptor (Fig. 4C) similar to the earlier discussed trial. The instrument manipulator was then used to advance the Teflon-tipped 41G cannula toward the macular haemorrhage using the robot’s z-axis control. The r-TPA delivery system was connected via the automated viscous fluid control port of the vitrectomy machine (Fig. 4D), allowing foot-controlled steady injection of the solution under direct intraoperative OCT visualization. Clear visualization of the retinotomy site, injection of r-TPA and bleb formation was possible with this technique (Fig. 5A). Fluid-air exchange was performed at the end of surgery with intravitreal injection of aflibercept and patients were advised to posture sitting upright at 45 degrees overnight to aid pneumatic displacement of thrombolysed blood.

Subretinal injections in this trial were all performed successfully except for one patient with a pre-existing posterior subcapsular cataract precluding a clear view of the cannula tip and therefore completed manually. Measurable trial outcomes were clinically and statistically similar between the robot-assisted and manual groups, although the results of this study should be considered within the context of its small cohort size. Of note, the median number of retinotomies was 1.0 in the robot-assisted, and 2.0 in the manual group. The median number of retinal microtrauma was also 0.0 in the robot-assisted group and 1.0 in the manual group. The subretinal injection time and total surgical time were similar in both groups. The median gain in visual acuity at post-operative month one was similar in both arms. Subretinal haemorrhage was successfully displaced at post-operative month 1 in all subjects except one patient in the manual group who likely experienced a rebleeding secondary to active disease (Fig. 3D). This trial demonstrated the ability of a telemanipulated robot to perform subretinal TPA injection safely and successfully inside the human eye. The key feature of the Preceyes system that assisted with subretinal injection was its ability to apply dynamic motion scaling, allowing precise and controlled movements with minimal retinal tissue injury, which was shown in the median number of retinal trauma in the robot group although it did not reach statistical significance.

### Retinal vein cannulation

The mainstay of treatment for retinal vein occlusion is targeted at the management of complications. Retinal vein endovascular surgery has been conceived and described, but cannulation of a vessel with a diameter of 150 µm followed by holding the needle tip in place for several minutes is considered an extremely difficult task [11]. Feltgen et al. described a retinal endovascular lysis with a fibrinolytic agent using manual vitrectomy in 2007 [38], and this surgical option had unacceptably high post-operative complications including neovascular glaucoma (46.2%), cataract formation (30.8%), retinal detachment (23.1%), and painful phthisis (15.4%). This proves that retinal endovascular surgery far exceeds the limitations of manual surgery.

Endovascular robotic surgeries have been performed and attempted in pre-clinical porcine models since 2016 using the Preceyes robotic micromanipulator. In one of the earliest preliminary studies, an in-vivo model for retinal vein cannulation was conducted in porcine eyes [33]. After inducing a retinal vein occlusion with laser and confirmation with fluorescein angiography, vein cannulation was performed with the Preceyes robotic system using a glass catheter tip. A large temporal sclerostomy was created, and a snug fit was achieved with the instrument manipulator. Several approaches were attempted, and the most successful approach was to position the tip of the needle over the middle section of the vein, providing slight indentation and then a robotically controlled piercing motion through the wall of the vein (Fig. 5C). The most successful location for cannulation were either associated with some tethering of the vessel to underlying structures or were adjacent to the occlusion. Successful cannulation using this approach with a balanced salt solution was seen in 9 out of 9 eyes. Another pre-clinic study was performed in porcine eyes with retinal vein cannulation and direct intraluminal injection of ocriplasmin [35]. Once again, a glass pipette with a terminal 30 µm outer diameter and a bevelled tip was used. Thrombus was released successfully in all eyes that had vein cannulation beyond the last venous branch point prior to the site of occlusion. In one eye, the catheter tip broke and re-insertion was attempted. The system proved capable of maintaining the cannular in position for up to 20 min. These pre-clinical studies showed that robot-assisted retinal vein cannulation with prolonged infusion time is technically feasible. Moving forward, further clinical trials are planned at the Oxford Eye Hospital using further optimized protocols and designs.

In-human in the context of phase I clinical trial has been performed with the robotic KU Leuven co-manipulator robotic system [32] in a small group of four patients with retinal vein occlusion in 2017 with intraluminal ocriplasmin injection. The needle tip is angulated to reduce the risk of double puncturing the vessel [23] and is covered by a retractable outer tube. Prior to the trial, ex vivo and in-vivo studies have been performed with the robotic system. In the in-vivo study, results showed successful cannulation in 15 out of 18 porcine eyes with holding the intravenous needle tip position for more than 3 min [11]. However, in one of the eyes, a torsional force transferred from the infusion line to the needle holder resulted in the breakage of the glass capillary. In the human trial, the surgeon was able to inject ocriplasmin into the targeted vein, with injection periods of up to 10 min. Further clinical outcomes are not yet published at the time of writing of this review.

### Integrating robotics into clinical practice

Robotic systems for vitreoretinal surgeries have huge potential for advancements and improvements, but given their relatively new nature, concerns arise regarding cost, surgical setup time, steep learning curve and training.

Expense is a major limiting factor with robotic systems, including annual maintenance, service contracts and cost of disposables [39]. Improved cost-effectiveness in terms of the usage of ophthalmic robotic systems needs to be in place, in order to move towards mainstream clinical use. However, some of these costs can be offset by improving outcomes, reducing complications, and intelligent design [40].

Operating room setup time can be significantly longer with robotic surgeries, and over 20 min have been documented in certain existing robotic systems [41]. In the earlier in-human trial with the Preceyes system, dissection of the retinal membranes took longer than with manual surgery [36]. However, with the most recent in-human randomized controlled trial performed [37], there were no statistically significant differences in surgical time between conventional manual surgery versus robotic-assisted surgery. This potentially goes to show that with increased practice and experience with robotic systems, surgical time can be reduced and given the benefits and stability of robotic surgery, the marginal increase in surgical time should not be clinically significant [37].

**Fig. 6:**
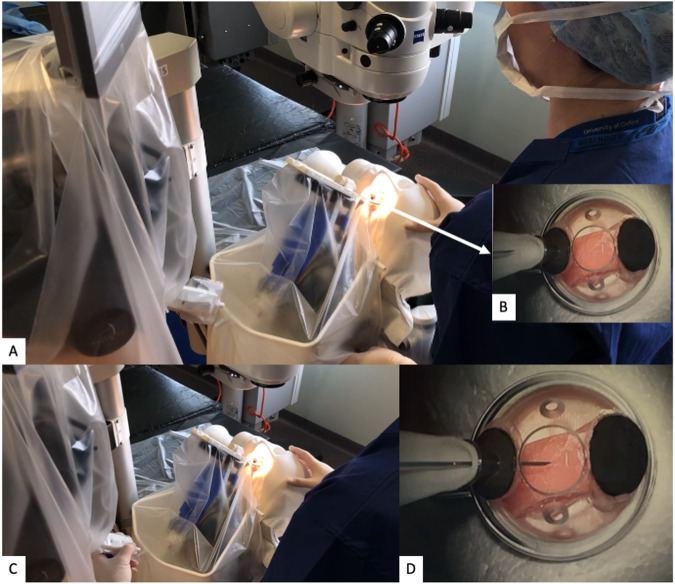
Surgical training using eye model system. **A**–**C** Extensive pre-training for surgeons using eye models with robotic system, with (**B**) showing a microscopic view of the eye model system with the fixed conical scleral adaptor. **D** Visualization of the advancing instrument in the model eye, which in this instance is a sharp pick for the initiation of an epiretinal membrane peel.

**Fig. 7:**
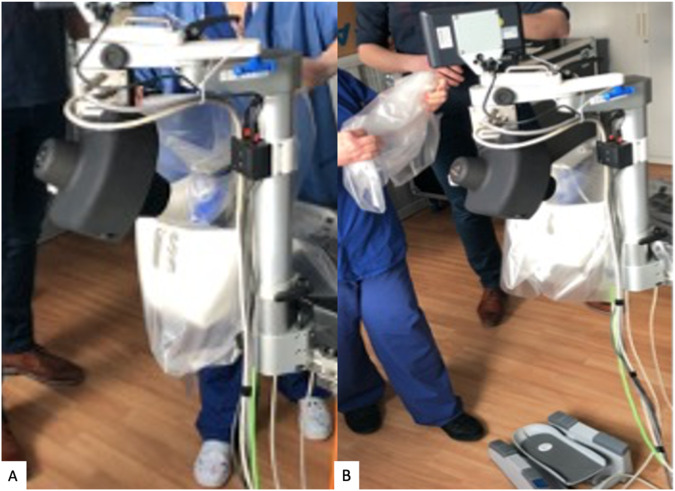
Multidisciplinary staff training. **A**, **B** Detailed multi-disciplinary training including all theatre staff involved in the entire process including the set-up of the robotic system.

The other limitation would be the learning curve for new robotic ophthalmic surgeons and surgical teams, and ensuring adequate training prior to the commencement of any robotic-related surgeries. There will need to be an overall training framework for robotic ophthalmic trainees and surgical teams, with a robotic surgical skills curriculum and a graduating model for granting robotic privileges [42]. In the conducted randomized controlled trials with the Preceyes system, extensive designated pre-training was done to the surgical team using eye models (Fig. 6). On top of that, all surgical theatre staff including surgeons, assistants, and scrub nurses underwent multi-disciplinary surgical training using model eyes to get used to the entire process involving the robotic surgical system (Fig. 7). This, in return, resulted in good safety profiles and outcomes in the trials. Streamlining the surgical training and familiarity with robotic systems will also shorten setup time which will ultimately improve efficiency during robot-assisted ophthalmic surgery.

Despite the limitations, there are definitely clinical applications where robotic-assisted systems are able to achieve what the manual surgical systems are not able to, as described earlier. Further refining of the robotic systems processes, robotic surgical training, and improving cost-effectiveness by intelligent design and deep learning will allow robotic-assisted intraocular surgery to gain acceptance into clinical practice. These include and are not limited to subretinal injection of therapeutic substances, complex retinal membrane peeling or delamination and endovascular surgery.

### The future applications

Robot-assisted vitreoretinal surgeries would potentially change the surgical treatment for advanced therapeutics such as for gene and cell therapy and optogenetics, and over the last decade our group has been at the forefront of this development [43]. There is a growing number of hereditary and degenerative diseases of the retina that are the target of pre-clinical or clinical research using both genetic vectors [44–52] and gene editing [53–56]. Optogenetics is a technique to control neural activity with light by the genetic introduction of light-sensitive proteins [57–60]. The expression of light-sensitive microbial opsins is a promising approach to restore vision in retinal degenerative diseases, and optogenetic tools can be genetically expressed in various sub-populations of retinal neurons using viral vectors [59]. The use of viral vectors is the mainstay of treatment moving forward for such degenerative conditions, and optimizing its delivery by reducing the loss of vectors from the intended target and reducing the risk of immunologic response [61]. Subretinal delivery via the trans-vitreal approach is the approach of choice for ongoing gene therapy clinical trials as it elicits less of an inflammatory response [44–52] compared to intravitreal injections [62] and suprachoroidal injections. Suprachoroidal injections also include a risk of choroidal haemorrhage, perforation, and no direct visualization of the amount injected. Subretinal injection does not seem to induce antibody production at low to median doses [63] as it is an immune-privileged space.

However, there are still certain challenges with subretinal injections of therapeutic substances. Delivering precisely to the subretinal space without breaching the Bruch’s membrane and the choroidal touch is challenging. The retina lacks elasticity and this implies that any lateral movement of the needle tip or any re-insertion attempts will be associated with a high risk of widening the retinotomy. This widened retinotomy will then result in reflux into the vitreous cavity, and this is common with current manual techniques, especially in pathological retinas which are thinned and atrophic [61, 64]. The Preceyes system user interface allowed surgeons to store the three-dimensional location of an instrument inside the eye. This unique ‘return to stored position’ feature could enable a needle tip to enter the same retinotomy twice without enlarging the size during a two-staged approach to subretinal injection [65], and this will in turn hugely help with the issue of reflux and ensuring the retinotomy remains in a standard size.

**Fig. 8:**
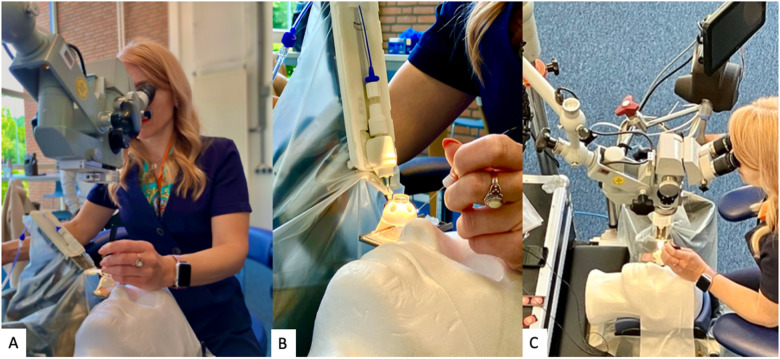
Optimizing robotic assistance for retinal surgery. **A**–**C** Ongoing collaborations between the vitreoretinal surgical team and the Preceyes team will allow further optimization of subretinal delivery of therapeutics.

The other challenge to subretinal delivery would be physiological hand tremors which are inadvertent especially when gene/cell solutions need to be delivered over a substantial period of time. Furthermore, static positioning for controlled delivery of vector or cell solutions into the subretinal space presents additional challenges and more pronounced movements [66]. Reflux into the vitreous cavity is another concern, resulting in loss of actual delivery of cells or vectors, and increased inflammation or epiretinal membrane formation [67–71] Direct volumetric measurements using intraoperative OCT showed that subretinal bleb size was on average 36% smaller than predicted [72]. This reflux can be minimized with prolonged retention of the needle tip in the subretinal space, and slow subretinal bleb formation with slow infusion of vectors or cells. This is limited to manual surgery and can be perfected with robotic-assisted systems. Robotic systems also provide the ability to dissect the surgical manoeuvre of subretinal delivery, by adjusting precisely the angle of penetration, depth, speed of injection and retinal contour [61]. Moving forward, it is necessary to demonstrate and optimize the microprecision robotic systems, especially in the context of subretinal therapeutics. There will be a need to establish the parameters required for the safe and effective delivery of various therapeutics to be eventually used clinically. We are working closely with the Preceyes surgical team with collaborations to allow us to further improve and optimize the subretinal delivery of therapeutics (Fig. 8).

As described earlier, retinal vein cannulation has huge promise for further advancements and optimization. This procedure has been shown to be too challenging with high complication rates when done manually [38] and hence robotic-assisted systems will fill in this gap. Most of the earlier pre-clinical models were performed with glass microneedles. This was developed following the specifications described by Pournaras et al. [73]. However, glass breakage has been seen in pre-clinical trials which are unacceptable in human eyes. A microfabricated needle-based cannulation system [74] was previously evaluated in porcine eyes, which showed a higher success rate (100%) in piercing and injecting a substance into the retinal vein than glass micropipette (40%). The main reason for failure was once again due to breakage of the glass micropipette. With this in mind, future endovascular cannulation will be evaluated at the Oxford Eye Hospital with the microfabricated needle to hopefully better the surgical outcomes.

## Conclusion

We have made significant progress with robotic-assisted intraocular surgeries [75], with several in-human trials showing good safety and effectiveness profiles [32, 36, 37]. Moving forward, there will be an ever-increasing need for higher precision in surgical procedures, as we dive into better outcomes for patients with conditions that are difficult to treat. Endovascular cannulation for central retinal vein occlusion and precise delivery of subretinal gene, stem cell, and optogenetic therapies are unachievable or surgically challenging with manual conventional vitreoretinal surgery. These are the exact areas that robotic-assisted devices will serve to fill in the gaps, make extra-delicate procedures possible and safe, and be synergistic with the advances made in these areas.
